# Pharmacodynamics, pharmacokinetics, interactions with other drugs, toxicity and clinical effectiveness of proton pump inhibitors

**DOI:** 10.3389/fphar.2025.1507812

**Published:** 2025-07-23

**Authors:** Łukasz Wołowiec, Joanna Osiak-Gwiazdowska, Albert Jaśniak, Michał Janiak, Lidia Wydeheft, Magdalena Łukasiak, Małgorzata Pellowska, Grzegorz Grześk

**Affiliations:** Department of Cardiology and Clinical Pharmacology, Faculty of Health Sciences, Ludwik Rydygier Collegium Medicum in Bydgoszcz, Nicolaus Copernicus University in Toruń, Bydgoszcz, Poland

**Keywords:** PPIs, pharmacokinetics, interactions, pharmacology, pharmacodynamics

## Abstract

The document comprehensively reviews proton pump inhibitors (PPIs), focusing on their pharmacodynamics, pharmacokinetics, drug interactions, toxicity, and clinical efficacy. PPIs irreversibly inhibit the H+/K+-ATPase enzyme in gastric parietal cells, effectively reducing gastric acid secretion. These drugs are widely prescribed for conditions like gastroesophageal reflux disease (GERD), peptic ulcer disease, eradication of *Helicobacter pylori* and as a prevention against bleeding from gastrointestinal tract. The review article highlights significant drug interactions associated with PPIs. Omeprazole, for instance, can interfere with the metabolism of clopidogrel, reducing its antiplatelet efficacy, which may have clinical implications. The article also discusses other drug interactions, including anticoagulants (e.g., warfarin), selective serotonin reuptake inhibitors (SSRIs), and immunosuppressive and chemotherapeutic drugs, as well as the side effects associated with taking PPIs. Long-term use of PPIs is linked to plenty of adverse effects, such as vitamin B12 and calcium deficiencies, which can lead to bone fractures. An increased risk of infections, including *Clostridium difficile* and small intestinal bacterial overgrowth (SIBO), is also noted. Cardiovascular risks, such as myocardial infarction and stroke, are observed in some patients on high-dose or prolonged PPI therapy. In rare cases, nephrotoxicity and hepatotoxicity are reported. Additionally, the document examines the potential role of PPIs in exacerbating certain cancers, such as gastric adenocarcinoma, and in influencing the severity of COVID-19 symptoms. PPIs are proven effective in treating GERD and preventing complications from nonsteroidal anti-inflammatory drugs (NSAIDs), particularly in reducing the risk of NSAID-induced ulcers. The document stresses the importance of understanding drug interactions and the need for individualized treatment to minimize adverse effects. Ongoing research into PPIs’ long-term safety and efficacy remains essential, particularly given their widespread use in clinical practice.

## 1 Introduction

The gastric acid secretion is involved in the digestion process, absorption of nutrients, and the prevention of infectious agents from transiting to the intestines. In the corpus of the stomach localized parietal cells are responsible for the production and release of HCl, which process is physiologically regulated by paracrine and neural pathways (Martinsen et al., 2019; Engevik et al., 2020).

The ultimate effector of the gastric acid secretion pathway is the proton pump, a membrane-bound enzyme called H+/K+-ATPase (H+/K+-adenosine triphosphatase), which is the target of medications used to treat gastric acid-related diseases (Sachs et al., 2006; El et al., 2018; Yao and Smolka, 2019; Strand et al., 2017; Del Re et al., 2021). By irreversible inhibition of the H(+)/K(+)-adenosine triphosphatase in cytoplasmic membranes of parietal cells of the stomach, Proton Pump Inhibitors (PPIs) are able to reduce the secretion of gastric acid (Sachs et al., 2006; El et al., 2018; Yao and Smolka, 2019; Srebro et al., 2022; Robinson and Horn, 2003; Ahmed and Clarke, 2023; Turshudzhyan et al., 2022).

PPIs are the main therapy for gastroesophageal reflux disease (GERD), which is one of the most commonly diagnosed gastrointestinal disorders in the United States with a prevalence of 20% (Cascella and Dulebohn, 2022). GERD is defined as abnormal reflux of gastric contents from the stomach into the esophagus or beyond including the oral cavity (Badillo and Francis, 2014). PPIs also have utility in treating of esophagitis, non-erosive reflux disease, peptic ulcer disease, prevention of nonsteroidal anti-inflammatory drug-induced ulcers, Zollinger-Ellison Syndrome and as a part therapy regimen for *Helicobacter pylori* infections (Ahmed and Clarke, 2023).

Over the past decade, many studies have evaluated the long-term PPI adverse effects (AEs) (Lehault and Hughes, 2017). Among the effects of long-term PPI intake is vitamin B12 deficiency, which can occur in long-term patients with nutrition disorders and the seniors in whom the risk of deficiency is 2–4 times higher (Novotny et al., 2019). Studies also report reduced calcium levels or even deficiency in patients with elevated stomach pH because calcium is not absorbed into the bloodstream and remains in the gastrointestinal tract for excretion. Without proper calcium levels in the body, osteoclasts and osteoblasts remain inactive, which hinders proper bone turnover (Lehault and Hughes, 2017). Studies have shown that long-term use of PPIs may increase the risk of bone fractures, but this requires further research (Aldulaijan, 2024; Kondapalli et al., 2023). Another reported side effect associated with long-term PPI treatment is an increased risk of infections - like, for example, small intestinal bacterial overgrowth (SIBO), nontyphoidal *salmonella* and *campylobacter* infections, *Clostridium difficile* infections (CDI), and pneumonia (Novotny et al., 2019). Increased risk of serious acute cardiovascular events, including acute myocardial infarction and stroke, has also been correlated with long-term or high-dose PPI treatment (Yibirin et al., 2021). Cardiovascular diseases along with renal failure (i.e., acute interstitial nephritis) and dementia are idiosyncratic side effects that are rare (Helgadottir and Bjornsson, 2019). The effects of short-term and long-term therapy are generally summarized in the table below (Table 1).

**TABLE 1:** The adverse effects of short-term and long-term therapy (Lehault and Hughes, 2017; Yibirin et al., 2021; Kitchlew, 2023).

S.No	Short-term adverse effects	Long-term adverse effects
1	Headache	Vitamin B12 deficiency
2	Rash	Calcium deficiency
3	Dizziness	Increased risk of infection
4	Gastrointestinal symptoms • Nausea • Abdominal pain • Bloating • Constipation • Diarrhea	Idiosyncratic side effects • Cardiovascular diseases (stroke, acute myocardial infarction) • renal failure • dementia

Among the six proton pump inhibitors (PPIs) approved by the FDA: omeprazole, esomeprazole, lansoprazole, dexlansoprazole, rabeprazole and pantoprazole, omeprazole holds a prominent position as one of the top ten most prescribed medications in the United States (Ahmed and Clarke, 2023; Turshudzhyan et al., 2022). In European Union esomeprazole and pantoprazole are the two PPIs which have been authorized by European Medicines Agency (EMA) (Annex, 2020; ROTS and Annex, 2019). Omeprazole, lansoprazole, dexlansoprazole and rabeprazole are authorized for use in the European Union in individual Member States via national procedures (ROTS and Annex, 2019). Furthermore there are PPIs available only in China, India and Korea such as ilaprazole, dexrabeprazole ad s-patoprazole tenatoprazole, AGN201904-Z, azeloprazole adanaprazole are in clinical trials (Ahmed and Clarke, 2023; Novotny et al., 2019).

This narrative review was based on a structured search of relevant literature across leading biomedical databases, primarily PubMed, supplemented by EMBASE, Web of Science, and Cochrane Library, which are widely recognized for their high-quality, peer-reviewed medical content. We considered articles published within the last 20 years, with a particular emphasis on literature from the past 8 years, which constitutes the majority of the references cited. This time frame was selected to ensure the highest possible reliability of our scientific work.

## 2 Pharmacokinetics

PPIs are characterized by relatively short half-life: 0.6–1.9 h. In contrast to the short half-life of PPIs the clinical effect of gastric acid suppression persists longer (Srebro et al., 2022; Turshudzhyan et al., 2022). Extended duration of clinical response after administration of these irreversible PPIs is the result of the time needed to form a new H+/K+-ATPase proton pump (54 h) (El et al., 2018; Turshudzhyan et al., 2022; Lima et al., 2021). Studies show that repeated administration leads to inhibition of more pumps, which enhance the suppression of gastric acid secretion (El et al., 2018; Lima et al., 2021).

Omeprazole, esomeprazole, lansoprazole, dexlansoprazole, rabeprazole and pantoprazole are metabolized by the liver enzymes, in majority by the isoenzyme CYP2C19, less by CYP3A4 but only two of them: omeprazole and esomeprazole are inhibitors of CYP2C19 which is significant for interactions between PPI and drugs metabolized by CYP2C19 (El et al., 2018). Rabeprazole is metabolized on non-enzymatic pathway (Turshudzhyan et al., 2022; Lima et al., 2021). Because of the impact of genetic and nongenetic factors, the response on treatment with PPIs may vary (Table 2).

**TABLE 2:** Pharmacokinetic of proton pump inhibitors (El et al., 2018; Strand et al., 2017; Srebro et al., 2022; Ahmed and Clarke, 2023; Turshudzhyan et al., 2022; Lima et al., 2021).

PPI	Omeprazole	Esomeprazole	Lansoprazole	Dexlansoprazole	Pantoprazole	Rabeprazole
Bioavailability (%)	30–40	64–90	80–85	NA	77	52
Time to plasma peak levels (h)	0.5–3.5	1.5	1.7	1–2	2–3	2–5
Volume of distribution (L/kg)	0.13–0.35	0.22–0.26	0.4	0.54	0.15	0.34
Half-life (T_1/2_)	0.5–1 h	1.3–1.6 h	1.6 h	1–2 h	1–1.9 h	1–2 h

PPI, proton pump inhibitor; NA, not available.

## 3 Pharmacodynamics

PPIs are acid-labile weak bases distributed as inactive agents and need low pH to start the inhibition of the H+,K+-ATPase. To prevent early activation within the stomach, it is necessary to safeguard them from the effects of gastric acid. This can be accomplished, for example, by enteric coating (Srebro et al., 2022; Proton Pump Inhibitors, 2012).

PPIs are benzimidazole derivatives, which optimal activity of PPIs is reached when they are administered in a fasting state. Patients are advised to take their PPIs on an empty stomach to achive maximum absorption of PPIs (Sachs et al., 2006; Strand et al., 2017; Robinson and Horn, 2003; Ahmed and Clarke, 2023). It is important to mention that administration with other anti-secretory agents may also have an impact on the activation and absorption of PPIs (Sachs et al., 2006).

All PPIs as an active sulfenic acid or sulfonamide bind covalently to cysteine residues on the H+/K+ ATPase and start to inhibit acid secretion until new pumps can be synthesized (Sachs et al., 2006; Strand et al., 2017; Ahmed and Clarke, 2023). There are some differences in localization of cysteine molecules: cysteine 813 is exposed on the luminal surface of the pump in a vestibule, cysteines 321, 813 and 822 are in the proton-transport domain of the H^+^K^+^-ATPase, cysteine 892 is localized on the external luminal surface. Cysteines 321 and 813 are in a luminal vestibule of the pump. Such differences in cysteine binding among the PPIs may underlie the differences among them in duration of inhibition of gastric acid secretion (Sachs et al., 2006; Shin and Sachs, 2008).

## 4 Interactions

### 4.1 General interactions with drugs of proton pump inhibitors (PPIs)

Interactions between medications are the result of two or more chemicals interacting with one another. This can alter the properties of the interacting compounds, change how they affect the human body, and increase the possibility of side effects.

Drug interactions come in three different forms:

1. A drug-drug interaction (DDI) is the result of two or more drugs interacting.
2. The reaction between a drug and a food or drink is known as a drug-food interaction.
3. Interaction between drug and condition: a reaction that happens when a drug is taken by a patient who has a specific medical condition (FDA, 2023).

Many medication interactions result from changes in drug metabolism. The cytochrome P450 (CYP) oxidases enzyme system is one significant system involved in metabolic drug interactions. Through receptor-dependent mechanisms, CYP enzymes can be transcriptionally activated by a variety of xenobiotics and endogenic substrates. One of the main mechanisms underlying metabolism-based drug-drug interactions is CYP enzyme inhibition. The CYP enzyme system can be inhibited or stimulated by many chemotherapeutic medications, which can lead to drug interactions (Manikandan and Nagini, 2018).

Omperazole, lansoprazole, esmoprazole, and pantoprazole are metabolized mainly by CYP2C19. The fraction of CYP2C19 metabolism for the mentioned drugs is 70%–80%. Rabeprazole is converted non-enzymatically to thioether; the role of CYP2C19 and CYP3A4 is significantly less in its hepatic metabolism. Tenatoprazole is metabolized by both CYP2C19, which promotes its hydroxylation at the C-5′ position, and CYP3A4 is mainly involved in the sulfoxidation reaction in which tenatoprazole sulfone is formed (El et al., 2018; Liao et al., 2023; Chevalier et al., 2023). There are a lot of important interactions which can cause serious health implications – in this article we tried to describe the most crucial ones.

### 4.2 Omeprazole – interactions with other drugs

Omeprazole is one of the earliest PPIs introduced to the pharmaceutical market (Proton Pump Inhibitors, 2012). It is an irreversible inhibitor of the H+/K + ATP-dependent proton pump of gastric parietal cells. Omeprazole is primarily metabolized by cytochrome CYP2C19 and CYP3A4 isoforms of the hepatic enzyme complex, which may result in many drug interactions (Shin and Sachs, 2008; Shah and Gossman, 2023; Dorji et al., 2022; Miedziaszczyk and Idasiak-Piechocka, 2023; Cartee and Wang, 2020). One of the more popular drug interactions is the interaction between omeprazole and clopidogrel, resulting from metabolism by the same enzymatic proteins: CYP3A4 and CYP2C19. Previous studies indicated the unfavorable combination of these two drugs, because omeprazole weakened the antiplatelet effect of clopidogrel, as it is the strongest inhibitor of the PPIs group of the previously mentioned CYP-450 isoforms (Juurlink et al., 2009). According to the recommendations of the European Society of Cardiology (ESC) and the American Heart Association (AHA), the patient should not be given omeprazole and clopidogrel at the same time, which is why doctors in clinical practice replace omeprazole with pantoprazole, avoiding a possible interaction between these drugs (Bang et al., 2020; Yan et al., 2022). However, recent studies have shown that this interaction (omeprazole and clopidogrel) when used concomitantly has no clinical significance. There is no clinically significant increase in clopidogrel concentrations, but investigators emphasize caution with concomitant use and individualization of treatment (Bouziana and Tziomalos, 2015; Abrignani et al., 2023; Nicolau et al., 2015). Moreover, the latest meta-analyses show that the simultaneous use of pantoprazole, lansoprazole or esomeprazole with clopidogrel increases the risk of cardiovascular complications, which have not been confirmed for omeprazole. Interactions between omeprazole and ticagrelor do not occur because ticagrelor does not require enzymatic conversion (Abrignani et al., 2023). Another antiplatelet drug is acetylsalicylic acid (ASA). The absorption of ASA depends on the pH of the stomach, hence long-term use of PPIs, including omeprazole, may weaken the effect of ASA (Scarpignato et al., 2016). The INR value is a great indicator to monitor among patients who take warfarin. Warfarin has been shown to interact with omeprazole, therefore INR monitoring should be recommended in these patients in order to implement possible treatment if the result is abnormal. Another anticoagulant drug is dabigatran, the absorption of which also depends on the pH of the stomach contents. Concomitant use of dabigatran and omeprazole reduces the bioavailability of dabigatran, but this is not clinically significant. The RE-DUAL study concluded that PPIs can be used regardless of the composition of triple anticoagulant therapy (Abrignani et al., 2023). Another group of drugs that have been shown to interact with omeprazole is the selective serotonin reuptake inhibitor: citalopram. The study was conducted in the Taiwanese community. When omeprazole and citalopram were used simultaneously, increased citalopram concentrations were found, which had a clinical significance in the studied patients. There was an increased risk of cardiovascular complications by prolonging the QT interval, which may lead to sudden cardiac arrest. Particular attention should be paid when using such drugs in older people (Wu et al., 2019; Sönnerstam et al., 2018). Interactions sometimes may be intensified by genetic changes in cytochromes. One example of a combination is the concomitant use of omeprazole and amlodipine, which may result in increased effects of amlodipine, leading to hypotension. Genetic testing of changes in cytochrome CYP3A4 may prove to be a new way to identify polymorphisms influencing the occurrence of interactions between drugs, which will be used in personalized diagnostics and patient treatment (Dorofeeva et al., 2019). Another example where CYP2C19 polymorphism was significant is the combination of fluvoxamine and omeprazole, which resulted in increased omeprazole concentrations, but only in patients with the CYP2C19*1 allele, which confirms that genotyping may avoid some drug interactions (Kamiya et al., 2019). Another clinically important combination is omeprazole and gliclazide, especially in patients with the CYP2C19 NM/RM/UM phenotype. In these patients, the risk of severe hypoglycemia increased threefold (Dujic et al., 2021). In addition, you can find many studies that analyze specific drug combinations and the occurrence of possible interactions between them. Researchers have proven that the concomitant use of omeprazole with the antifungal drug voriconazole resulted in an increase in this drug because omeprazole inhibited CYP2C19, which is responsible for the metabolism of voriconazole (Tian et al., 2021). A similar effect can also be obtained by taking tacrolimus and omeprazole together, which may lead to an increase in tacrolimus plasma concentration and, consequently, intensify its side effects, e.g., nephrotoxicity. Plasma levels of this drug should be monitored (Miedziaszczyk and Idasiak-Piechocka, 2023). It has been demonstrated that omeprazole concentrations in plasma may increase by up to 80% when administered concomitantly with adavosertib (Någård et al., 2023). Studies were also carried out which showed no change in the pharmacodynamics and pharmacokinetics of the drug, despite the interaction. An example of such a combination is the use of omeprazole and pyrotinib, or the use of omeprazole and padacitinib or Roxadustat (Li et al., 2022a; Mohamed et al., 2020; Groenendaal-van de Meent et al., 2018). In addition, attention should be paid to non-pharmacological interactions. The food consumed by the patient may have a significant impact on the absorption of drugs. The cite study proved that taking the drug on an empty stomach is more beneficial for the patient for protecting the gastric mucosa than taking the drug after a meal, because meal in the stomach limits the absorption and effect of the drug (Ochoa et al., 2020).

### 4.3 Pantoprazole – interactions with other drugs

Pantoprazole is another drug from the group of PPIs which is mainly metabolized by CYP3A4 (Proton Pump Inhibitors, 2012; Bernshteyn and Masood, 2023). Pantoprazole is a weak inhibitor of CYP2C19, therefore it often is an alternative to omeprazole. However, the latest research also took into account the dose and method of drug administration, which influenced the occurrence or severity of drug interactions. This study showed that intravenous administration of omeprazole or pantoprazole had a significant impact on the pattern of voriconazole concentration, which resulted in an increase in the toxicity of this drug,^56^/80/ however, it is indicated that pantoprazole is less clinically important than omeprazole in increasing the concentration of voriconazole, especially with oral administration, but caution should be exercised when using these drugs simultaneously (Blanco et al., 2020). Many studies prove that the inhibitory effect of pantoprazole on CYP2C19 is lower than that of omeprazole, because when pantoprazole and tacrolimus, everolimus, or sirolimus were used simultaneously, the minimum concentrations of tacrolimus, everolimus, or sirolimus were not affected (Bremer et al., 2018). It was similar when pantoprazole and ripretinib were used simultaneously – no clinical significance (Li et al., 2022b). A study was conducted which also showed no effect on the concentration of the drugs: palbociclib or ribociclib with the simultaneous use of pantoprazole (Odabas et al., 2023). In clinical practice, it is possible to replace omeprazole with pantoprazole while using clopidogrel in order to avoid drug interactions and thus weaken the antiplatelet effect. It was shown that pantoprazole did not affect the effect of clopidogrel (Westergaard et al., 2021; Farhat et al., 2019; Wang et al., 2015; Lin et al., 2020). Additionally, it was confirmed that the use of pantoprazole in a patient taking dual antiplatelet therapy did not weaken the effect of the drugs or affect the aggregation of platelet (Choi et al., 2017). It has been proven that the use of elbasvir, grazoprevir or famotidine is safe in combination with pantoprazole (Feng et al., 2017). A study was conducted among patients using one of these drugs: citalopram, escitalopram or sertraline, which showed that pantoprazole did not affect the concentrations of these drugs, which is evidence of a minimal inhibitory effect on CYP2C19, which metabolizes SSRI drugs. Additionally, it was shown that concomitant administration of omeprazole or esomeprazole with SSRIs interacted by inhibiting CYP2C19, resulting in an increase in SSRIs (Gjestad et al., 2015). Particular caution should be exercised when using diclofenac and pantoprazole together, as an interaction between these drugs may occur, as they are metabolized by the same 2C subgroup, diclofenac by CYP2C9, and pantoprazole by CYP2C19 (Ertekin et al., 2015). Patients who take dipeptidylpeptidase-4 inhibitors (DPP-4Is) should pay special attention to the level of glycemia when using pantoprazole, because as a result of the interaction between these drugs, the risk of hypoglycemia increases up to 2-fold (Ray et al., 2021). Pantoprazole should be taken before a meal, as food may delay the absorption and bioavailability of the drug (Ochoa et al., 2020).

### 4.4 Esomeprazole – interactions with other drugs

According to studies, esmoprazole in high doses can strongly inhibit CYP2C19, but weakly inhibits CYP3A4 and slightly induces CYP1A2 (Kaartinen et al., 2020). Esomeprazole, like other PPIs, is a commonly used drug which carries the risk of multiple adverse interactions. During treatment with esmoprazole, gastric pH increases, it can be hypothesized that this may affect the absorption of pH-sensitive drugs such as digoxin and ketoconazole (Andersson et al., 2001). Staying with anticoagulants, however, a large prospective study examined the effect of PPIs including esmoprazole on acenocoumarol treatment, specifically whether concomitant PPIs use was associated with an increased risk of hypercoagulability during acenocoumarol maintenance treatment and whether the effect was modified by alleles of CYP2C19 variants. Esomeprazole doubled the risk of hypercoagulability (Teichert et al., 2011). Esomeprazole interacts with compounds metabolized by CYP2C19, as demonstrated by the examples of phenytoin and R-warfarin, although it was emphasized that these interactions did not reach clinical significance (Wedemeyer and Blume, 2014). Looking at the above from the other side, meta-analyses show a protective effect of PPIs when it comes to gastrointestinal bleeding (GIB) with certain anticoagulants (Table 3) (Bang et al., 2020). Interaction studies of esomeprazole and SSRIs showed that sertraline levels were significantly higher in patients treated with esomeprazole (+38.5%; P = 0.0014) (Gjestad et al., 2015). With that said, other proton pump inhibitors (e.g., omeprazole, lansoprazole and pantoprazole) did not appear to affect the pharmacokinetics of sertraline. In the case of benzodiazepines, it has been proven that multiple doses of esomeprazole increased the concentration of diazepam and decreased its elimination, and this had its symptoms clinically as impaired motor coordination and alertness (Wedemeyer and Blume, 2014). Interaction studies of PPIs have also focused around the statins, because by competing with such isoforms, PPIs can reduce the metabolism of statins, causing them to be more effective in lowering LDL. Nevertheless, PPIs have so far not been found to interact with statins (Barkas et al., 2015). On the other hand there was a study in which esomeprazole used with atorvastatin was attributed to the occurrence of rhabdomyolysis in a patient, as the pharmacokinetic profiles of these agents suggest that a possible contributing factor to this reaction was the inhibition of P-glycoprotein (PGP) by esomeprazole, altering the normally significant first-pass clearance of atorvastatin (Sipe et al., 2003). There are also studies determining the effect of the use of the mentioned PPI on liver iron conection (LIC) where, in an intention-to-treat analysis, a significant effect of esomeprazole treatment was observed: the reduction in LIC (delta LIC) was significantly greater after 1 year of esomeprazole compared to 1 year of placebo (mean difference in LIC reduction 0.55 mg Fe/g dw; 95% CI [0.05 to 1.06]; p = 0.03). In the case of esomeprazole, the interactions are numerous, prompting extra attention when prescribing it (van Vuren et al., 2022).

**TABLE 3:** The protective effect of taking PPIs in preventing GIB during the use of selected anticoagulants (Bang et al., 2020).

Anticoagulant	Comorbidities or concomitant factors	Prevention of GIB by PPI1 Yes/insignificance/no
Dicumarin	High baseline risk of gastrointestinal injury	Yes
Warfarin	Past history of GIB, old age, comorbidities, *H. pylori* infection, and co-administration of antiplatelet agents	Insignificance (only in one study)
Dabigatran	Past history of GIB, old age, comorbidities, *H. pylori* infection, and co-administration of antiplatelet agents	No

PPIs, proton pump inhibitors; GIB, gastrointestinal bleeding.

### 4.5 Rabeprazole - interactions with other drugs

Rabeprazole is another drug from the group of PPIs that will be discussed in our study. Rabeprazole seems to have a weaker potential for interactions with drugs and less impact on the microsomal activity of the liver, even in cirrhosis patients (Wedemeyer and Blume, 2014; Rocco et al., 2021).

Compared to omeprazole or pantoprazole, rabeprazole drug interactions have been examined less thoroughly, as seen by the high number of unidentified outcomes (Wedemeyer and Blume, 2014). When it comes to rabeprazole and food Ochoa et al. (2020) proved that the absorption of rabeprazole is delayed by food, which can cause variations in Tmax (time to peak drug concentration). In certain subjects, this delay can reach up to 20 h. So it might be preferable to administer PPIs during a fast since food increases the variability of PPIs absorption and delays it.

#### 4.5.1 Rabeprazole and clopidogrel

PPIs were known for its conflicting information about the impact of drug interactions, but clinical outcomes have mostly come from non-randomized observational research (Scott et al., 2014). When it comes to recent studies researches proved that rabeprazole does not have significant DDI with clopidogrel (Przespolewski et al., 2018). When taken in conjunction with clopidogrel, the risk of major adverse cardiovascular events was not increased by rabeprazole. When co-prescription with dual antiplatelet therapy (DAPT) is indicated, rabeprazole appears to be a good option due to its minimal drug interaction profile and optimal acid suppression (Przespolewski et al., 2018; Dalal et al., 2023). Another randomized prospective study strengthen the findings of rabeprazole about its not significant effects on the dual antiplatelet therapy involving clopidogrel and aspirin. They measured the risk of high platelet reactivity (HPR) when co-prescribing clopidogrel and rabeprazole vs. clopidogrel and famotidine. Primary endpoint was 5 μM ADP-induced platelet aggregation at 30-day follow-up. HPR was defined as 5 μM ADP-induced PA > 46%. Between the famotidine and rabeprazole groups, the frequency of HPR was comparable (20.5% vs. 15.4%; P = 0.555). Ultimately, the antiplatelet effect of rabeprazole was found to be similar to that of famotidine when used as an addition, even in patients who were sensitive to clopidogrel (Ahn et al., 2020).

#### 4.5.2 Rabeprazole and immunosuppressive drugs and cytostatics

Some PPIs can have DDI with tacrolimus, a very commonly prescribed drug, especially for kidney transplant patients. Concha et al. (2023) documented in a case raport that rabeprazole has a small effect on the tacrolimus blood concentration and is safe to co-prescribe, for example, in patients after kidney transplant. Some research proves that rabeprazole can have some interactions between certain anticancer drugs.

Ribociclib and palbociclib are indicated for the treatment of HR-positive, HER2-negative, locally advanced or metastatic breast cancer. Studies showed that rabeprazole significantly prolongs the metabolic elimination of ribociclib and palbociclib. It is recommended that physicians beware when providing PPIs to these patients as a result (Li J. et al., 2019). Presented scientific proof points out the possibility of PPIs, including rabeprazole, inhibiting P-glycoprotein. Therefore, in order to prevent side effects, monitoring is necessary when co-administering those two drugs (Desai et al., 2023).

Another cytostatic drug that has been tested for interaction with rabeprazole is oxaliplatin. Oxaliplatin is used to treat colorectal cancer, treatment of stage III colon cancer after complete resection of the primary tumor, metastatic colon and rectal cancer. Hashizume et al. (2022) looked into whether PPIs lessen oxaliplatin’s antitumor effect. Cancer cells take up oxaliplatin through the organic cation transporters OCT1-3. PPIs, on the other hand, inhibit OCT1-3’s activity. The study demonstrated that, even in the event that oxaliplatin and PPIs interact, clinical doses of PPIs were thought to have little bearing on oxaliplatin’s antitumor effect. Another study examined the effects of rabeprazole on the pharmacokinetics and antitumor efficacy of capecitabine and its metabolites. For capecitabine and its three metabolites, there were no notable impacts of rabeprazole on the area of the plasma concentration-time curve split by capecitabine dosage. The suppression of colon cancer cell growth by the corresponding capecitabine metabolites was not affected by rabeprazole. The pharmacokinetics of capecitabine are unaffected by rabeprazole (Shaik et al., 2019).

Gao N. et al. (2022) screened 114 drugs to determine the potential interaction with osimertinib based on the rat liver microsome (RLM) reaction system. Osimertinib is a drug used to treat non-small cell lung cancer. Researches proved that AUC (the area under the plasma drug concentration-time curve which reflects the actual body exposure to drug after administration of a dose of the drug) and peak concentration of osimertinib significantly decreased following oral co-administration of rabeprazole, however, they significantly increased upon intraperitoneal injection of osimertinib. When combined, their findings show that proton pump inhibitors significantly affect osimertinib’s behavior, offering fundamental information for the accurate administration of osimertinib.

#### 4.5.3 Rabeprazole and clobazam

Clobazam is used for its anxiolytic effect, and as an adjunctive therapy in epilepsy.

Pasupuleti et al. (2020) set out a study to determine PPIs including Pantoprazole, Esomeprazole, and Rabeprazole impact on clobazam levels in plasma.

Rabeprazole and esomeprazole have a less significant impact on the combining with PPIs on the plasma levels of clobazam than do pantoprazole. When it is necessary to combine clobazam with PPIs, it is best to choose rabeprazole or omeprazole due to their lower effect on plasma drug levels.

### 4.6 Lansoprazole - interactions with other drugs

Lansoprazole is the second drug from the PPIs group introduced to the market (Proton Pump Inhibitors, 2012). Lansoprazole is a prodrug that is used, for example, in gastric ulcer disease. A stereoisomer was produced - dexlansoprazole, which has been used since 2009 (Jewell, 2019). /93/ Lansoprazole undergoes interactions characteristic of the PPIs group. Examples include interaction with clopidogrel or SSRI drugs: escilatopram and citalopram. Particular caution should be exercised when these drugs are used concomitantly, as increases in escilatopram or citalopram concentrations may result in prolongation of the QT interval (Cena et al., 2020). When vorticonazole and a PPI drug are used concurrently, the concentration of vorticonazole should be monitored because it may increase, increasing the toxicity of this drug to the patient. However, the use of lansoprazole and vorticonazole in patients is relatively safe, unlike omeprazole or esomeprazole (Mafuru et al., 2021). Concomitant use of quizartinib and lansoprazole did not reduce the effects of the drugs (Li J. et al., 2019). It has been proven that long-term treatment with PPIs, including lansoprazole, does not affect the effect of ribociclib (Del Re et al., 2022).

### 4.7 Tenatoprazole - interactions with other drugs

Tenatoprazole is a drug prospective as a proton pump inhibitor (PPI). It is being tested clinically as a possible treatment for peptic ulcers and reflux oesophagitis (Li et al., 2013).

While all PPIs are effective in reducing acidity during the day, tenatoprazole, a new medication, can be used to treat acid throughout the night (Koyyada, 2021).

It is conceivable because of its extraordinarily long duration of effect, which stems from its plasma half-life, which is approximately seven times longer than that of other PPI medications (Le et al., 2023).

However, tenatoprazole is not yet approved by the FDA for clinical use (Koyyada, 2021).

Because of this, there is not much evidence of interactions other than this original paper by Nies et al. (2011) where they focused on the role of drug transporters in pharmacokinetics. Since metformin is a substrate for each of the uptake transporter proteins OCT1, OCT2, and OCT3, the focus of the current study was on these proteins. The most important finding of their research was that all tested PPIs, including tenatoprazole, significantly inhibited the metformin uptake transport for each of the three OCT proteins that were examined. Furthermore, researchers were able to demonstrate that none of these PPIs are the three OCT transport proteins’ substrates. Together, these findings showed that PPIs, including tenatoprazole, were a significant class of medications that block the OCT-mediated transport of metformin. But it was pointed out that more clinical research is necessary to clarify the *in vivo* significance of this recently identified *in vitro* drug-drug interaction with reference to drug disposition and/or pharmacodynamic effects in metformin-treated patients (Table 4.).

**TABLE 4:** Summary of pharmacokinetic drug interactions.

Interaction between	Citalopram	Clopidogrel	Diazepam	Ivabra-dine	Levo-thyroxine	Metoprolol	Tacrolimus	Warfarin
Omeprazole	↑ Unfavorable	↓ Unfavorable	↓ Unfavorable	None	Unknown	None	Unknown (maybe ↑) 58	↓ Unfavorable
Pantoprazole	None	None	None	Unknown	None	None	None	None
Esomeprazole	↓ Unfavorable	↓ Unfavorable	↑ Unfavorable	Unknown	Unknown	Unknown	Unknown	None (maybe unfavorable)
Lansoprazole	↑ Unfavorable	None	None	None	Unknown	Unknown	↓ Unfavorable	↑ Unfavorable
Rabeprazole	Unknown	None	None	Unknown	Unknown	Unknown	None	None
Dexlansoprazole	Unknown	None	None	Unknown	Unknown	Unknown	Unknown	None
Tenatoprazole	Unknown	Unknown	Unknown	Unknown	Unknown	Unknown	Unknown	Unknown

↑ – increase in drug concentration due to interaction.↓ – decrease in drug concentration due to interaction.

## 5 PPIs interaction with programmed death protein-1/ligand-1 used in cancer treatments

Wu et al. evaluated the impact of omeprazole and other PPIs (pantoprazole, lansoprazole, esomeprazole, rabeprazole and dexlansoprazole) on the survival of cancer patients treated with programmed death protein-1/ligand-1 (PD-1/L1) inhibitors. Eight studies involving 4,869 patients were included. The results of this meta-analysis showed that omeprazole use was associated with a shorter overall survival. In solid cancer patients receiving PD-1/PD-L1 inhibitors, the use of PPIs was associated with a 43% increased risk of death. Still additional clinical trials and experimental investigations are required to validate the impact and underlying mechanism of PPI on exacerbating the clinical outcome of PD-1/L1 inhibitor (Wu et al., 2022) (Table 5).

**TABLE 5:** Characteristics of studies included in meta-analysis (Wu et al., 2022).

Study	Treatment	Cancer	PPI type
Chalabi 2020	Atezolizumab	NSCLC	Omeprazole, pantoprazole, lansoprazole, esomeprazole, rabeprazole, and dexlansoprazole
Hopkins 2020	Atezolizumab	Urothelial cancer	Omeprazole, pantoprazole, esomeprazole, lansoprazole, rabeprazole, and dexlansoprazole
Hopkins 2021	Atezolizumab	NSCLC	Omeprazole, pantoprazole, esomeprazole, lansoprazole, rabeprazole, and dexlansoprazole
Svaton 2020	Nivolumab	NSCLC	Omeprazole, pantoprazole, and lansoprazole

PPI, proton pump inhibitor; NSCLC, non-small-cell lung cancer.

## 6 The impact of PPIs treatment on the course of SARS-CoV-2 infection

The pandemic caused by the SARS-CoV-2 virus was a huge challenge for health protection. During the COVID-19 pandemic, many studies have been conducted on the impact of various factors that may have contributed to the increased risk of illness, severe symptoms of infection, complications and mortality. One of the possible routes of infection with the SARS-CoV-2 virus is the oral route. PPIs are one of the most frequently prescribed drugs, hence the interest of many researchers in the possible impact of the use of these drugs on the course of COVID-19 disease. The effect of PPIs is to increase the pH of the stomach, which promotes the development of the microbiome in the stomach. Based on studies and meta-analyses, it was found that the use of PPIs contributed to the increase in the severity of COVID-19 symptoms and secondary infection. The probable cause is the previously mentioned increase in gastric pH and, as a result, increased virus multiplication, which translated into an increased cytokine storm, leading to more severe symptoms presented by the patient. It was found in this meta-analysis that the use of PPIs did not translate into an increase in mortality due to SARS-CoV-2 virus infection (Shokri et al., 2023). Veettil et al. (2022) showed that the use of PPI has been connected to a higher risk of COVID-19-related mortality as well as other associated side effects, such as severity and longer hospital stays. However, they indicate the need for further research to confirm this theory (Fatima et al., 2022).

In July 2020, a large study found that PPI users were more likely to report a positive test for SARS-CoV-2.6 In contrast, Lee et al. (2021) found that current PPI use was associated with an increased risk of serious COVID-19 outcomes, but not with a risk of infection. Similarly, Zhou et al. (2021) reported an association with serious outcomes, including the need for intensive care unit (ICU) admission, intubation or death (Israelsen et al., 2021).

Meta-analysis by Yan et al. showed that there is no association between use of PPI and higher risk of infection with COVID-19. In this publication the analysis of two original studies showed that PPI use was associated with increased risk of secondary infection by *Clostridium difficile*, *Campylobacter*, *Salmonella* and also with a higher risk of nosocomial pneumonia in patients in intensive care units (Yan et al., 2022). Also Veettil et al. (2022) found that previous use of PPIs may put patients at risk for problems such as an increased risk of C. diff., which could have a significant impact on the course of treatment.

The associations observed between PPI use and adverse COVID-19 outcomes should be interpreted with caution. Further randomized controlled trials or well-adjusted prospective cohorts are warranted to better delineate these relationships.

## 7 Toxicity of PPIs

Over the past few decades, the utilization of proton pump inhibitors has significantly risen, giving rise to concerns regarding their toxicity and reported adverse effects associated with them. Still, future studies are needed to fully explain the toxicity of long-term-use of PPI.

It has been shown that omeprazole, especially for long-term use, may induce DNA damage by inducing oxidative stress, apoptosis and necrosis. All of the available PPIs can induce chromosomal damage (Paz et al., 2020).

Many studies have indicated that hypoacidity and hypergastrinemia can elevate the chances of developing gastric cancer (Fossmark et al., 2015). Proton pump inhibitors (PPIs) can potentially exhibit tumor-promoting effects through elevation of plasma gastrin levels (Bridoux et al., 2022). A recent report in a Swedish population has highlighted that individuals who use PPI medications are more prone to developing gastric adenocarcinoma (Brusselaers et al., 2017). During pregnancy, esomeprazol can cause harm to the liver, leading to a decrease in the levels of aspartate aminotransferase (AST) and alanine aminotransferase (ALT) enzymes (Thomas et al., 2016). Chronic use of omeprazole has been associated with detrimental impacts on the liver and kidneys, such as hepatotoxicity and nephrotoxicity (Paz et al., 2020). Patients with liver disease may face a heightened susceptibility to hepatotoxicity, potentially resulting in the development of carcinogenic effects induced by hypergastrinemia, affecting liver cells (Fossmark et al., 2015). It also has been shown that chronic utilization of PPIs in individuals suffering from chronic kidney disease heightens the risk of mortality and progression of the kidney disease (Giusti et al., 2021).

Currently available meta-analyses indicate an association between PPI use and, among others, an increased incidence of gastric atrophy and gastric cancer, and between PPI use and an increased risk of chronic kidney disease and end-stage renal disease. However, further research is needed to confirm these correlations (Gao H. et al., 2022; Vengrus et al., 2021; Li et al., 2017).

## 8 Effectiveness of group of PPI drugs

IPP as mentioned earlier in this article are frequently prescribed drugs. It is worth leaning into their effectiveness. In the case of GERD, with the use of PPIs overall, there was a reduction in complaints in about 70% of patients (Liakakos et al., 2010). However, efficacy in GERD strongly depends on the age group to which we refer, because, for example, clinical trials reveal that PPI therapy is not an effective treatment for common infant GERD-associated symptoms (Higginbotham, 2010). In the case of eosinophilic esophagitis (EoE), high-dose PPI treatment was effective in half of the EoE patientsin cohort study of 236 adult with EoE (Frandsen et al., 2021). The importance of PPIs is also emphasized in preventing complications from NSAID use. Namely, PPIs have been shown to reduce NSAID-induced indigestion (Leontiadis et al., 2007). It is emphasized that currently the best way to reduce iatrogenic ulcer bleeding from gastric and duodenal ulcer is the administration of anti-ulcer drugs, including proton pump inhibitors (PPIs) (Kim et al., 2019). As the article shows PPIs find effective use in many diseases and conditions. In Tables 6, 7, we discuss the clinical problems resulting from the use of PPIs and clinical indications for PPI: their corresponding benefits and potential risks (Tables 6, 7).

**TABLE 6:** Clinical problems resulting from the use of PPIs.

Clinical concern	Mechanism of interaction	Clinical implications
Reduced efficacy of clopidogrel	Omeprazole or ezomeprazole CYP2C19 inhibition	Reduction in antiplatelet activity
Vitamin B12 deficiency	Increased gastric pH-impairs absorption	Anemia, neuropathy, cognitive decline
Hypomagnesemia	Reduced Mg absorption	For example,: Cramps, arrhythmia
Methotrexate interaction	Concomitant use of methotrexate (primarily at high doses) with PPIs may decrease methotrexate clearance	Myelotoxicity, mucositis
C. difficile infection	Reduced gastric acid protection	Diarrhea and later complications
Fracture risk	Decreased calcium absorption	Osteoporosis
Reduced iron absorption	Impaired conversion of ferric ion (Fe3 +) to divalent ion (Fe2 +), which occurs in an acidic environment	Iron-deficiency anaemia, Iron therapy failure
Warfarin interaction	Interact with omeprazole	INR variability, bleeding risk
Reducing the antifungal activity of ketoconazole	Increased gastric pH-impairs absorption	Treatment failure
Levothyroxine interaction	Increased gastric pH-impairs absorption	TSH fluctuation

**TABLE 7:** Clinical indications for proton pump inhibitors: Corresponding benefits and potential risks.

Clinical aspect	FDA-approved uses	Potential benefits	Potential risks	Monitoring/Clinical considerations
GERD (Gastroesophageal Reflux Disease)	Short- and long-term treatment of symptomatic GERD	Symptom control, healing of esophagitis	Rebound acid hypersecretion, potential masking of malignancy, adverse reactions of PPIs	Symptom assessment after 8 weeks of treatment
Erosive Esophagitis/Barrett’s Esophagus	Healing and maintenance of erosive esophagitis (FDA)	Reduction of dysplasia risk in Barrett’s esophagus	Long-term nutrient malabsorption, microbiota alterations	Endoscopic surveillance; continue if histologic/clinical benefit confirmed
NSAID/Antiplatelet Prophylaxis	Prevention of gastric ulcers associated with NSAID therapy (FDA for omeprazole, esomeprazole)	Protection against gastric or duodenal ulcer formation, Reduction of GI bleeding risk	Drug interactions (e.g., with clopidogrel), possible renal implications	Assessing the need for NSAID therapy, reducing the dose of PPIs
*H. pylori* Eradication Therapy	*H. pylori* eradication	Enhances antibiotic effectiveness	Minimal risks due to short-term treatment	Stop PPI after eradication confirmed
Zollinger–Ellison Syndrome	Treatment of peptic ulcers	Healing of peptic ulcers	Adverse reactions of PPIs	Monitor electrolytes and bone health during chronic high-dose use
Fracture Risk	Not an FDA indication	Not Applicable	Increased risk of bone fractures with long-term use due to the risk of developing osteoporosis	Consideration of DEXA scan in older adults or high-risk patients; supplement calcium + vitamin D if needed
Infection Risk	Not an FDA indication	Not Applicable	Risk of *C. difficile* infection, community-acquired pneumonia	Watch for unexplained diarrhea; avoid unnecessary antibiotic or PPI overlap
Nutrient Deficiency Risk	Not an FDA indication	Not Applicable	Vitamin B12, magnesium, and iron malabsorption	Yearly blood work in long-term treatment or elderly patients
Drug Interactions	Not an FDA indication	Not Applicable	CYP2C19-mediated (e.g., clopidogrel, methotrexate), digoxin potentiation	Prefer pantoprazole if interactions are a concern; reconcile medications regularly
Deprescribing Considerations	Not an FDA indication	Avoiding polypharmacy	Rebound hyperacidity, poor tapering can worsen outcomes	Taper slowly, consider using H2 blockers during transition off PPI

## 9 Future research directions

One direction for further research may be the association between long-term PPI use and the development of dementia. Further well-designed cohort studies with control for confounding factors are needed to clearly determine the effect of PPIs on the risk of dementia, as there is currently insufficient evidence (Li M. et al., 2019; Ahn et al., 2023). In addition, a systematic review found that in 20 of 21 meta-analyses, the use of proton pump inhibitors (PPIs) was consistently associated with an increased risk of gastric cancer, with reported relative risks ranging from 1.3 to 2.9. However, the available studies were of limited statistical power, highlighting the need for further studies that take into account factors such as *Helicobacter pylori* infection, PPI dosage, and duration of therapy to better understand the mechanisms underlying the increased risk of gastric cancer (Brusselaers et al., 2025; Abrahami et al., 2022). Potassium-competitive acid blockers (P-CABs), such as vonoprazan, may be a safe and effective alternative to PPIs, but further study is needed, especially considering the long-term use of these drugs in the context of adverse effects (St. Onge and Phillips, 2023). Effective educational and intervention strategies should be developed to reduce PPI overuse and monitor their long-term impact on patient health (Dharmarajan, 2021).

## 10 Conclusion

Knowledge of drug interactions is essential for proper treatment and to avoid possible adverse drug reactions. Drugs should be selected with great care when used by the patient, as the intensification of effects resulting from the interaction of certain groups of drugs may be life-threatening. Sometimes a drug can be replaced with another drug from the same class to avoid adverse interactions, as this review confirms. Some of the interactions are highly complicated, and many other processes are still unknown, which is why continuous research on interactions between drugs, especially frequently used ones such as PPIs, is very important for the health and life of patients.

Long-term use of PPIs must be tailored to the clinical context and in accordance with the indications that are approved by the US Food and Drug Administration (FDA) for the treatment of various gastrointestinal conditions associated with hyperacidity. Considering the comorbidities, different drug combinations can be considered, taking into account the potential for interactions. In elderly patients or those taking multiple medications, unnecessary continued use of PPIs may contribute to the risk of polypharmacy. In elderly patients or those taking multiple medications, it is recommended to choose a PPI with a lower potential for drug interactions, such as pantoprazole or rabeprazole. Similarly, patients with chronic kidney disease, osteoporosis or vitamin B12 deficiency may be susceptible to the adverse effects of long-term PPI use, therefore the need for medication should be reassessed, with the aim of discontinuing the medication or using the lowest effective dose, as some conditions, such as Barrett’s esophagus or eosinophilic esophagitis, justify continuous PPI therapy due to their protective benefits. In patients with mild or remitting symptoms (e.g., GERD or functional dyspepsia), discontinuation may be considered with appropriate monitoring. In addition, when PPIs are used for gastroprotection (e.g., during dual antiplatelet therapy), the choice of the appropriate PPI (such as pantoprazole to avoid CYP2C19 inhibition) is crucial. Patients taking warfarin for atrial fibrillation and concomitant Duodenal Ulcer should be wary of omeprazole and lansoprazole, which interact with this drug. Pantoprazole or dexlansoprazole would be a safer option. If ivabradine is necessary, omeprazole or lansoprazole can be used. Additionally, in patients taking citalopram for depression, pantoprazole would be a good choice if a PPI is necessary.

Clinical decision-making should also take into account the risk of infections such as Clostridioides difficile, especially in elderly or hospitalized patients. Ultimately, a risk-benefit assessment for the individual patient should guide both continuation and discontinuation of PPIs. Another aspect that requires reassessment of the appropriateness of PPI use is emerging anemia, as the causes to be eliminated include the use of PPIs, which due to adverse effects can lead to reduced vitamin B12 availability. The indications should be monitored, as in patients without a clear indication for therapy, PPIs should be discontinued. An example of short-term use (up to 8 weeks) are GERD symptoms, after which the efficacy of treatment and further management should be assessed.

Direct comparative studies of different PPIs are needed to establish relative efficacy and safety profiles, and studying genetic factors influencing PPI metabolism can lead to personalized therapy, optimizing efficacy while minimizing adverse effects. Potassium-competitive acid blockers (P-CABs), such as vonoprazan, have shown promise as effective alternatives to PPIs, offering rapid and sustained acid inhibition. Further studies are needed to assess their long-term safety and potential role in clinical practice.

In summary, although PPIs are effective, their use should be individualized, regularly assessed, and based on emerging evidence to ensure optimal patient outcomes. Knowledge of drug interactions is essential for proper treatment and to avoid possible adverse drug reactions. Drugs should be selected with great care when used by the patient, because the intensity of the effects resulting from the interaction of some drug groups can be life-threatening. Sometimes a drug can be replaced by another drug from the same class to avoid adverse interactions, as confirmed by this review. Some interactions are highly complex, and many other processes are still unknown, so continued research on drug interactions, especially frequently used ones such as PPIs, is very important for the health and life of patients.
